# The Hurried Life

**Published:** 1905-09

**Authors:** Thomas Darlington

**Affiliations:** Health Commissioner of Greater New York


### The Hurried Life.

### Business Man and Working Man Are Equal Sufferers.

By THOMAS DARLINGTON, M. D.,
Health Commissioner of Greater New York.

[*From The Tribune Sunday Magazine.*]

"YOUR money or your life" seems to be one of those phrases which the world is not permitted to forget. No longer shouted by bold highwaymen on lonely roads through midnight darkness, it is now being cried in open daylight to all men by the vital statistics of Greater New York. And the modern cry is far more serious than the old one, carrying with it, as it does, a penalty which no man who is sacrificing health for wealth can possibly hope to escape.

Interesting indeed are these mathematical and undeniable witnesses. The moral which they impress is important to all and particularly important to two widely separated classes in the community, viz.: The Business Man and the Working Man. Irish-Americans and German-Americans will also find in them food for thought if not for alarm. Looked at from the standpoint of nationalities, these two classes are marked as the special victims of the two diseases which our present mode of life is developing most prominently.

That, as a people, we are always in a tremendous and unnecessary and injurious state of hurry many may not know. In comparison with the people of other countries, however, we are, in this respect, ludicrous. A young Greek just arrived in New York called on me one day and said that there was great excitement on Broadway; all the people were rushing somewhere; but he could not find out what it was about. It slowly dawned upon him after a few days that the people were always rushing somewhere and that what they were about was merely their private business.

Another foreigner newly arrived in Brooklyn saw all the people running for a New York ferry boat and promptly joined in the chase. Breathless and panting, he reached the gate a moment after it was closed and became greatly excited. In broken English he offered a handsome sum of money to anyone who would row him across the East river. He was gazed at in astonishment by the people; but he insisted that it was vitally important for him to reach New York. When told that the bridge was open and that there would be another boat in two minutes he gasped in amazement. He had supposed, from the mad rush of the people, that it was the last boat of the day. That another one was available in two minutes left him wordless in astonishment. "It is you who are crazy; not I," he said.

Watch men run at full speed up the stairs to the elevated railway, watch them run along the platforms, watch them rush breathlessly across subway stations as if their lives depended upon catching that particular train instead of the one three minutes later, and the foreigner's charge will have a certain value. Hurry with us has become a mania. And the two diseases which hurry and the hurried life has developed, especially, are a warning to many men and women that if they do not promptly begin to take life in a more leisurely way they must pay the severest of all penalties for their folly.

The increase in deaths from heart disease is a warning to all business men and all society women. This increase has been startling and out of all relative proportion. In one month of the present year New York has had one hundred and twenty-five deaths from this cause, as against only fifty-six deaths from the same complaint in the corresponding month of last year. Along the line of records for thirty years we find that while the general death rate in proportion to population has steadily lowered, heart disease and Bright's disease, to which it is closely related, have increased three hundred per cent. The only means of stopping this increase and the increasing ratio is a general campaign of education, which will reach the individuals most concerned, because every man's life is in his own hands and he will not live it properly unless he is taught to do so. To business men, society women, working men, Irish-Americans and German-Americans this article therefore is addressed primarily as a beginning of this necessary campaign.

If one started out to drive a horse thirty-five miles in one day he would not whip him over the last five miles. Common sense and a decent consideration for the horse would withhold the whip during the last five miles of the journey. Yet the New York business man shows less common sense and less consideration for himself in the later and most dangerous period of his life than he would show to a horse. In the great pressure of life in a money-mad nation, a nation which is prone to sacrifice all the best values in life for values which are imaginary, the business man works up to forty or forty-five to the full potential energy of his constitution.

When the vital climax is reached and the descending period of vital energy,—the great law of Nature which can never be evaded,—sets in, instead of working less he works more. His business has expanded, his responsibilities are greater than ever, his goal has become higher, the demands upon his time and energy have increased instead of diminished. He has less energy to work with, he stores less during sleep, he begins to spend his vital capital instead of the interest on his capital, and begins to complain of his health. Stimulants of all kinds are restored to—he begins to whip the horse—the signs of old age, white hair, diminishing powers, come so quickly that they seem magical, the heart which is feeling the unnatural demand most heavily deteriorates most rapidly and under some sudden strain it gives way and the man dies perhaps twenty years before his allotted time.

In England he retires from business at fifty. He thenceforth takes only a mild supervisory interest in the conduct of affairs and devotes himself mainly to collecting china or old prints, or growing prize peaches or preposterous chrysanthemums. Preposterous chrysanthemums are only a mild occupation after the stress and struggle of the arena, but they serve. The art of life lies in getting all there is out of it—all the juice there is in the orange. And for this purpose any one fad may serve as well as another, a hale and hearty old age being the practical proof of wisdom and success.

Much the same may be said of the smart society woman. The incessant round of social demands, nightly and daily, coupled with the imperative demands of her household and private affairs, leave her no time for proper sleep. When she goes to the country, after the season, she does not rest and exercise, as do the majority of Englishwomen, but continues the same excess of social indulgences. The first sign of failing health is failing beauty, since beauty is only the external proof of health. The strain is as great with her as with the man, and the victims are nearly as many.

Before proceeding to give the proper daily régime of a business man's life, a word must be said generally as to worry.

Worry, as well as hurry, is the great evil of the day. The former may be a necessary result of the latter, and worry in its last analysis may be merely the reflection of an unhealthy physical state; but it is an evil in itself which should be fought against especially. Nothing diminishes the action of the heart and places a greater burden on the arterial system than worry. It creates toxins of its own in the system whose elimination is a heavy added strain upon the vital energy.

The first rule for all persons engaged in the hurried life should be "Don't worry!" If the answer is "How can I help it, under the circumstances?" then alter the circumstances. If your business or social commitments are such as to compel worry, diminish them to a rational amount. You must choose between this and health, between a rational life or an early death. When such people realise that the early death is certain they will find that a change in their mode of life is far easier than they supposed it to be.

The business man should begin the day with a good breakfast. His rapid brain in its constant work demands food to supply its energy, and if food energy is not available he will always be tempted to supply the lack with expensive stimulants—expensive in their vital and constitutional effect. This breakfast, varying according to taste, should include fruit, a cereal, eggs and bacon or chop or steak, and coffee which is one-half milk and cream. In the forenoon he should never fail to drink at least one full glass of pure water. There are any number of good waters on sale now, and I hope to see the time when New York's own water supply will be as perfect in character as any of those now on the market.

His lunch should always be moderate. If it is too heavy the heavy demand upon his attention when he returns to his desk, by forcing the blood to his brain, will handicap his digestion of a too-ample meal and lead in time to chronic indigestion, a complaint which he, least of all men, can afford to entertain. A single dish easily digestible should suffice for lunch. His dinner should be eaten not later than seven o'clock at night, and should not be heavy—should not include more than three courses.

And now comes the period of the day in which most of the injury is done and in reference to which advice cannot be impressed too deeply. This concerns the importance of sleep and the importance of preparation for sleep.

The brain and nervous system of the body are a storage battery. Fully occupied with bodily demands during waking hours, sleep gives them the opportunity to store the necessary force for the coming day. Perfect sleep means that they can undisturbed devote themselves completely to this work. Partial sleep, partial unconsciousness, means interference with the storage process and diminution of the amount of energy stored. The brain is as fully under the dominion of habit as the hand or any set of muscles that have been trained to any habitual coördination. As a mother's first thought is her child, a business man's first thought is his business, and the man's brain is always willing and eager to think about business when the man wants to. Only complete exhaustion will make it break this rule. Thoughts of business therefore are the things which tend most greatly to deprive the business man of healthy and perfect sleep, and he should begin to eliminate them long before the sleeping hour. He should leave his business behind him when he leaves his office. If he does not let his brain quiet down and seek milder and "sleepier" subjects of attention, it will not do its proper work after his eyes are closed.

The after-dinner hours therefore should be hours of preparation for sleep. Black coffee should never be drunk after dinner nor in the evening by any person who wishes to sleep soundily. It is the most excellent of all methods of banishing sound sleep. One cigar is all that any man should allow himself in the evening. This is not the time for the hard and active thought which the cigar assists. Any amusement that is not too exciting, any pleasant entertainment that causes him to forget business, is good. Bridge whist and poker are not good. They keep brain and heart going under the strain that he has come to love as an opium-taker loves his drug. Quieting down is the process he should seek. Light exercise before retiring is an excellent habit. The ideal exercise is a walk for less than a half mile. Failing this, dumb-bells or one of the lifting-machines will pull the blood from his brain to the muscles, burn up any excess of nourishment at dinner and prepare him for ideal sleep, the ideal storage of force.

A business man who will thus take his sleep seriously may work to a hale old age. He is balancing his vital account healthily at the close of every day. The business man who is in the rush of Wall street all day and continues the rush over the same subjects of attention, cigars and hard thinking at the Waldorf-Astoria in the evening will not do so for many years. Watch the café there, and in a few years you will see his hair whiten, and all the signs of old age appear, until one evening he is missing, somewhere between fifty and sixty, and his tale is told. He is a fool: he has lost the game; and you have won because you have lived and he has not. This is the only sensible way to look at the great game called Life. To realise the value of healthy sleep and so devote the evening hours to pleasant preparation for healthy sleep will add many years to any business man's life.

When we come to the working man, we find a different state of affairs from an entirely different cause. We find the remarkable fact that his Utopia, the Utopia of eight hours and higher wages, is serving him ill. We find him much less healthy, enjoying life less and dying earlier than when he worked longer hours and earned lower wages. We find that he now has the money and time to overindulge himself and that overindulgence is killing him off through Bright's disease three times as rapidly as was the case twenty years ago.

The excessive rise of deaths from Bright's disease of three hundred per cent. is about parallel with that in heart disease, and the working class are most prominently affected by it. As has been said, however, the two complaints are closely related, and the rich, next to the workers, are the greatest sufferers numerically from overindulgence.

Whenever any epidemic occurs among the working classes we find a large, a disproportionate, number of deaths on Monday. The obvious cause is that the working man plays too hard on Sunday. He tires out not only himself but his wife and all the children with the long day of pleasure-seeking, overdrinking and overeating on the one day in his week which is devoted to enjoyment.

This brings up the important question of alcoholism, a potential factor in the great rise in deaths from heart disease as well as Bright's. In Manhattan and The Bronx, from January 1 to May 31 of this year, the death rate from heart disease to the ten thousand was: Irish, 15.71; Germans, 14.49; Americans, 5.08.

The great majority of victims among the Americans were business men and society women, as is shown by the localities. Among the Irish and Germans the rate is distributed more evenly over the city. But the enormous disproportion shows the terribly injurious part which is played in the strain of metropolitan life by alcohol. The two peoples which are the great consumers of alcohol show a vast disproportion in the deaths from heart disease and Bright's disease.

A curious change has come over the community in the last twenty years in this respect. According to the hospital records, whisky is doing much less harm than formerly, and the malt liquors much greater harm. The sclerotic conditions which are sure signs of the former have given place to degenerate arterial conditions which are the equally certain marks of the latter. But the injury which alcohol is doing is so apparent as to need no particular emphasis. The moral of the whole subject is temperance—temperance in work, temperance in play, temperance in eating and drinking. This has been preached so often, however, and from so many sources that the oft-repeated phrase will fall on dull and unresponsive ears. The practical necessity is education. We are a practical people, and every man who realises that in his hurry and worry he is paying much and getting little is cheating himself out of his best years, and his best good in life will eventually change his mode of living. Older communities have learned the lesson, and hale old Englishmen and hearty old Frenchmen are taking an active part in their national and municipal affairs in a way that would discourage what has been called the Oslerian idea. Three-score years and ten are every man's right, and only he himself can cheat himself of his heritage. The evils which accompany the abundance of the rich are far more apparent today than when they were first pointed out by Ecclesiastes, and if the sage had added that "All is vanity except good health" he would have added a valuable precept to his many wise saws.

Further special education is needed by every business man, such as is described by the Japanese proverb: "At forty every man is a physician or a fool." This means that by the time he is forty every sensible man has a practical working knowledge of that machine, his body, upon whose proper care his success, health and happiness depend.

Furthermore, as a nation, we should take play more seriously. We should devote more time to it. Our extraordinary folly in this respect was well exemplified some years ago when a Pittsburg manufacturer sent an agent to Belgium to obtain as many skilled iron and steel workers as were available. They were offered such unheard-of wages as compared with those which they were getting that they appointed a committee to investigate. The committee came over, looked into the lives of the Pittsburg steel workers and went back. They reported: "It is all work; no pleasure. The Americans are crazy." There were no orchestral societies, no hours spent in public gardens, no thought of the humble pleasures which can accompany life in the humblest menage. It was all work, and nothing else was thought of.

And what the Belgian committee found to be true in one industry is true of our life generally. We get little or nothing out of it, when we should get as much as others do and can, by taking its responsibilities more lightly and its pleasures more seriously.

